# Quality of Life Evaluation Before and After Pulmonary Lobe Resection for Benign Diseases: A Comparative Study Among Patients with Tuberculosis, Bronchiectasis, and Benign Lung Nodules

**DOI:** 10.3390/diseases12120307

**Published:** 2024-11-30

**Authors:** Alin Nicola, Tamara Mirela Porosnicu, Sorina Maria Denisa Laitin, Cristian Oancea, Emanuela Tudorache

**Affiliations:** 1 Department of Thoracic Surgery, “Victor Babes” University of Medicine and Pharmacy Timisoara, Eftimie Murgu Square 2, 300041 Timisoara, Romania; alin.nicola@umft.ro; 2 Doctoral School, “Victor Babes” University of Medicine and Pharmacy, Eftimie Murgu Square 2, 300041 Timisoara, Romania; 3 Department of Anesthesia and Intensive Care, “Victor Babes” University of Medicine and Pharmacy Timisoara, Eftimie Murgu Square 2, 300041 Timisoara, Romania; mirela.porosnicu@umft.ro; 4 Discipline of Epidemiology, “Victor Babes” University of Medicine and Pharmacy Timisoara, Eftimie Murgu Square 2, 300041 Timisoara, Romania; 5 Center for Research and Innovation in Precision Medicine of Respiratory Diseases, “Victor Babes” University of Medicine and Pharmacy, Eftimie Murgu Square 2, 300041 Timisoara, Romania; oancea@umft.ro (C.O.); emanuela.tudorache@umft.ro (E.T.)

**Keywords:** quality of life, pulmonary lobe resection, benign lung disease, tuberculosis, bronchiectasis, benign lung nodules, SF-36, WHOQOL-BREF

## Abstract

Background: Pulmonary lobe resection is a common surgical intervention for various benign lung diseases, including tuberculosis (TB), bronchiectasis, and benign lung nodules. While immediate clinical outcomes are well documented, the impact on patients’ quality of life (QoL) remains less explored. This study aims to evaluate QoL before and after pulmonary lobe resection over a 6-month period among patients with benign lung diseases. Objectives: To assess and compare changes in QoL among patients undergoing pulmonary lobe resection for TB, bronchiectasis, and benign lung nodules, and to identify factors influencing QoL outcomes. Methods: This prospective study included 84 patients who underwent pulmonary lobe resection for benign lung diseases, divided into three groups: TB (n = 22), bronchiectasis (n = 31), and benign lung nodules (n = 31). QoL was assessed using the SF-36 and WHOQOL-BREF questionnaires preoperatively and at 6 months postoperatively. Anxiety and depression were evaluated using the Hospital Anxiety and Depression Scale (HADS), and perceived stress was measured using the Perceived Stress Scale (PSS-10). Statistical analyses included paired *t*-tests, ANOVA, and Pearson’s correlation, with *p*-values < 0.05 considered significant. Results: At 6 months postoperatively, all groups showed significant improvements in physical and mental QoL scores (*p* < 0.05). The TB group exhibited the greatest improvement in physical health domains, while the bronchiectasis group showed significant enhancement in social functioning. Anxiety and depression scores decreased significantly in all groups, with the TB group showing the most substantial reduction (*p* < 0.01). Perceived stress levels also decreased across all groups. Comparisons revealed that the TB group had significantly higher QoL improvement compared to the other groups (*p* < 0.05). Conclusions: Pulmonary lobe resection for benign diseases significantly improves QoL over a 6-month period, particularly in patients with TB. The findings suggest that surgical intervention not only alleviates physical symptoms but also enhances psychological well-being. These results underscore the importance of considering QoL outcomes in the management of benign lung diseases requiring surgical intervention.

## 1. Introduction

Pulmonary lobe resection is a common surgical procedure performed for both malignant and benign lung diseases. While significant attention has been given to oncological outcomes following lung resections, less emphasis has been placed on the quality of life (QoL) outcomes for patients undergoing surgery for benign conditions. Understanding the impact of surgery on QoL is crucial, as it encompasses physical, psychological, and social well-being, which are important determinants of overall health [1].

Tuberculosis (TB) remains a major global health challenge, with an estimated 10 million new cases and more than 1.5 million deaths worldwide in 2020 [2]. In some cases, pulmonary TB leads to extensive lung damage requiring surgical intervention, especially in multidrug-resistant cases [3]. Surgical resection in TB can remove diseased tissue, reduce bacterial load, and improve respiratory function [4].

Bronchiectasis is characterized by irreversible dilation of the bronchi, leading to chronic cough, sputum production, and recurrent respiratory infections [5]. The global prevalence of bronchiectasis is increasing, particularly among older adults [6]. When medical management fails to control symptoms, surgical resection of the affected lobe can offer symptom relief and improve QoL [7].

Benign lung nodules are often incidental findings on imaging studies, with an estimated prevalence of up to 50% in high-risk populations [8]. While many lung nodules are asymptomatic and benign, some require surgical removal due to size, growth, or uncertain diagnosis to exclude malignancy [9]. The psychological burden of uncertainty and the potential risk of malignancy can significantly impact patients’ QoL [10].

Despite the clinical benefits of pulmonary lobe resection in these conditions, the impact on patients’ QoL before and after surgery remains underexplored. Previous studies have primarily focused on postoperative complications and survival rates after lobe resections [11,12,13]. However, QoL assessments provide valuable insights into the patients’ perspectives on their health status and the effectiveness of the surgical intervention for lung masses [14].

Assessing QoL involves evaluating various domains, including physical functioning, mental health, social relationships, and environmental factors [15]. Instruments like the Short Form-36 Health Survey (SF-36) and the World Health Organization Quality of Life-BREF (WHOQOL-BREF) are validated tools commonly used in clinical research [16,17]. Understanding changes in QoL can guide clinicians in preoperative counseling and postoperative care planning [18]. Therefore, this study aims to evaluate and compare the QoL before and after pulmonary lobe resection over a 6-month period among patients with TB, bronchiectasis, and benign lung nodules. By identifying factors influencing QoL outcomes, we hope to enhance patient care and inform clinical decision-making. 

## 2. Materials and Methods

### 2.1. Study Design and Ethical Considerations

This research was structured as a cross-sectional study spanning 24 months, from June 2022 through June 2024. It was carried out within the Thoracic Surgery Department of Victor Babes Hospital for Infectious Diseases and Pneumology in Timisoara and the Marius Nasta Institute in Bucharest, Romania. Prior to the commencement of the study, ethical approval was secured from the Institutional Review Boards of the hospitals. The study was conducted in adherence to the ethical principles outlined in the Declaration of Helsinki [19]. To participate in the study, all participants were required to provide written informed consent, affirming their voluntary involvement and understanding of the study procedures.

### 2.2. Study Participants, Inclusion and Exclusion Criteria

For the current study on the outcomes of pulmonary lobe resection, a total of 84 continuous patients were selected and enrolled. The inclusion criteria were set to comprise adults aged between 18 and 75 years. Secondary inclusion criteria were the selection of study groups: tuberculosis (TB), bronchiectasis, and benign lung nodules. Additional criteria were that all patients must require pulmonary lobe resection. Furthermore, participants needed to have the ability to understand and complete the required study questionnaires, along with a willingness to provide informed consent and comply with the procedural demands of the study.

Conversely, several exclusion criteria were established: (1) individuals with a history of malignant lung diseases; (2) previous lung surgery; (3) significant comorbidities that could impair quality of life, such as uncontrolled cardiovascular diseases or severe psychiatric disorders. Additionally, candidates unable to complete the follow-up assessments due to logistical or health reasons were also excluded. 

Upon screening, the patients were categorized into three distinct groups according to their diagnosed conditions. The first group comprised 22 patients with pulmonary TB who required lobe resection due to either cavitary lesions or multidrug resistance. The second group included 31 patients suffering from localized bronchiectasis that remained unresponsive to medical therapy and thus needed surgical intervention. Lastly, another group of 31 patients was identified with benign lung nodules; these patients required surgical resection for either diagnostic confirmation or to provide symptomatic relief. 

All participants in the study were diagnosed with benign lung diseases; thus, they had not undergone prior chemotherapy or radiotherapy. Patients with tuberculosis presented with resistant or recurrent manifestations of the disease, thereby necessitating lobectomy. Similarly, individuals with bronchiectasis and those with benign lung lesions had not received any specific treatment prior to their inclusion in the study.

### 2.3. Data Collection and Outcome Measures

Baseline demographic and clinical data were gathered from each participant in the study. Key information such as age, gender, smoking status, existing comorbidities, and results from pulmonary function tests (FEV1, FVC) were recorded to establish patients’ profiles. Additionally, quality of life (QoL) was assessed using two instruments: the SF-36 and WHOQOL-BREF questionnaires. These assessments were conducted both preoperatively and then again six months after the surgical procedure. 

The SF-36 Health Survey and the WHOQOL-BREF questionnaire were designed to measure multiple facets of QoL. The SF-36 Health Survey evaluates eight domains that encompass physical functioning, role limitations due to physical health problems, bodily pain, general health perceptions, vitality, social functioning, role limitations due to emotional problems, and mental health. On the other hand, the WHOQOL-BREF assesses four broader domains: physical health, psychological health, social relationships, and environment. 

Additionally, psychological well-being was evaluated using the Hospital Anxiety and Depression Scale (HADS) [20] and the Perceived Stress Scale (PSS-10) [21]. The HADS is focused on identifying the presence and severity of anxiety and depression symptoms, while the PSS-10 quantifies perceived stress levels. To ensure the integrity and impartiality of the data, these assessments were conducted by trained research assistants who were not involved in the direct care of the patients. 

### 2.4. Statistical Analysis

The sample size for the study was calculated based on the goal of detecting a medium effect size (Cohen’s d = 0.5) with 80% statistical power at a 0.05 significance level. This resulted in a requirement of at least 22 patients per group.

The statistical analysis for this study was executed using SPSS version 26, provided by IBM Corp., Armonk, NY, USA. Continuous variables were analyzed as follows: normally distributed data were represented as means ± standard deviation (SD), and non-normally distributed data were expressed through medians and interquartile ranges (IQR). Categorical data were summarized as frequencies and percentages. For analysis within groups, comparing preoperative and postoperative conditions, paired *t*-tests or Wilcoxon signed-rank tests were used depending on the data distribution. Between-group comparisons to detect differences among different patient categories were conducted using one-way ANOVA with post hoc Tukey tests for normally distributed data or Kruskal–Wallis tests for non-parametric data. Additionally, categorical variables were analyzed using chi-square tests.

Further statistical analyses included correlation analyses to explore relationships between various clinical and demographic variables, utilizing Pearson’s or Spearman’s correlation coefficients as appropriate for the data type. A *p*-value of less than 0.05 was set to determine statistical significance. The sample size calculation aimed to detect a medium effect size (Cohen’s d = 0.5) with an 80% power at a 0.05 significance level, necessitating at least 22 patients per group. 

## 3. Results

Table 1 shows the baseline demographic and clinical characteristics of the patients across the three groups. The mean age was comparable among the groups (*p* = 0.512), indicating no significant age differences that could confound the QoL outcomes. Gender distribution was similar, with a slight male predominance in all groups (*p* = 0.891). Smoking status was also comparable, with no significant differences in the proportions of current smokers, ex-smokers, and non-smokers (*p* = 0.927). Comorbidities such as hypertension and diabetes mellitus were evenly distributed among the groups (*p* = 0.823). Baseline pulmonary function, assessed by FEV1 (% predicted), was similar across groups (*p* = 0.814), indicating comparable respiratory function prior to surgery.

Table 2 presents the baseline QoL and psychological scores. SF-36 Physical Component Summary (PCS) and Mental Component Summary (MCS) scores were similar across the groups (*p* = 0.512 and *p* = 0.678, respectively), indicating comparable physical and mental health status before surgery. WHOQOL-BREF domain scores were also comparable, with no significant differences observed in physical health, psychological, social relationships, or environment domains (*p* > 0.6). HADS scores for anxiety and depression were similar among the groups (*p* = 0.821 and *p* = 0.915), indicating that patients had comparable levels of psychological distress preoperatively. PSS-10 scores, reflecting perceived stress levels, were also similar (*p* = 0.772).

The TB group showed significantly higher SF-36 PCS and MCS scores compared with the bronchiectasis and benign nodules groups (*p* = 0.013 and *p* = 0.045, respectively), indicating greater improvements in physical and mental health. WHOQOL-BREF scores in the physical health and psychological domains were also significantly higher in the TB group (*p* = 0.004 and *p* = 0.011), suggesting better QoL outcomes. No significant differences were observed in the social relationships and environment domains (*p* > 0.4). HADS scores for anxiety and depression were significantly lower in the TB group (*p* = 0.022 and *p* = 0.037), indicating reduced psychological distress. PSS-10 scores were significantly lower in the TB group (*p* = 0.019), reflecting decreased perceived stress levels (Table 3).

Table 4 compares preoperative and postoperative scores within each group. All groups showed significant improvements in SF-36 PCS and MCS scores (*p* < 0.001), indicating enhanced physical and mental health post-surgery. The TB group exhibited the largest mean differences in both PCS and MCS scores (+11.6 and +11.5, respectively). HADS anxiety and depression scores decreased significantly in all groups (*p* < 0.001), with the TB group showing the greatest reductions (−4.3 for both). PSS-10 scores also decreased significantly across all groups (*p* < 0.001), indicating reduced perceived stress.

**Figure 1:**
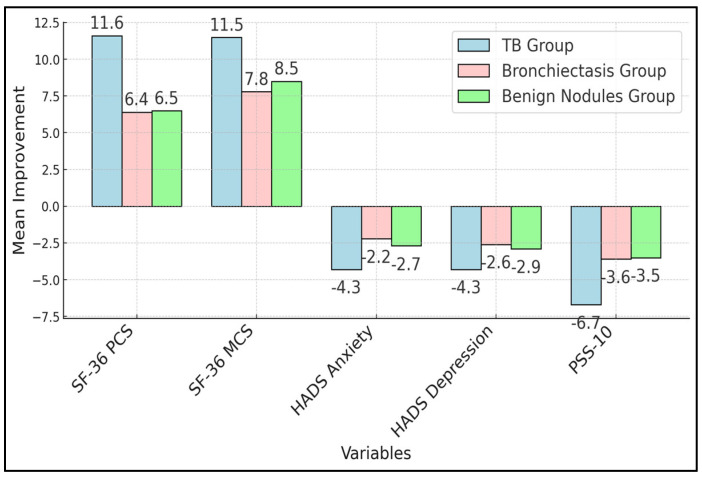
Between-group comparisons of QoL improvements.

The TB group showed significantly greater improvements in SF-36 PCS and MCS scores compared to the bronchiectasis and benign nodules groups (*p* = 0.002 and *p* = 0.013, respectively). Similarly, reductions in HADS anxiety and depression scores were significantly greater in the TB group (*p* = 0.004 and *p* = 0.006). The TB group also had a significantly larger decrease in PSS-10 scores (*p* = 0.001), indicating greater stress reduction. Post hoc analyses confirmed that the TB group’s improvements were significantly higher than those of the other two groups, as presented in Figure 1.

Table 5 shows the correlation between QoL improvement and clinical variables across all patients. A significant negative correlation was found between age and SF-36 PCS improvement (r = −0.35, *p* = 0.001), indicating that younger patients experienced greater physical health improvements. A significant positive correlation was observed between baseline FEV1 (% predicted) and SF-36 PCS improvement (r = +0.42, *p* < 0.001), suggesting that better preoperative lung function was associated with greater QoL gains. No significant correlations were found between smoking status or comorbidities and QoL improvement (*p* > 0.1).

Table 6 summarizes postoperative complications and outcomes. The rates of postoperative complications were low and did not differ significantly among the groups (*p* = 0.815). Complications included prolonged air leak, wound infection, and pneumonia. The length of hospital stay was slightly longer in the TB group (7.5 ± 1.8 days) compared to the bronchiectasis (6.8 ± 1.5 days) and benign nodules groups (6.5 ± 1.4 days), but this difference was not statistically significant (*p* = 0.089). There were no deaths within 30 days post-surgery in any group.

## 4. Discussion

### 4.1. Literature Findings

This study evaluated the impact of pulmonary lobe resection on QoL among patients with TB, bronchiectasis, and benign lung nodules over a 6-month period. The findings demonstrated significant improvements in both physical and psychological domains of QoL across all groups. Notably, patients in the TB group experienced the most substantial gains, suggesting that surgical intervention may have a more pronounced effect in this population.

The greater improvement in the TB group could be attributed to the resolution of active infection and the removal of diseased tissue, leading to enhanced respiratory function and symptom relief [22]. This aligns with previous studies indicating that surgery can be an effective adjunct to medical therapy in multidrug-resistant TB [23]. The significant reductions in anxiety, depression, and perceived stress in the TB group may reflect psychological relief from overcoming a serious infectious disease [24].

In the bronchiectasis group, improvements in QoL were observed but were less pronounced compared with the TB group. This may be due to the chronic and progressive nature of bronchiectasis, where surgical resection of localized disease may not fully alleviate symptoms or halt disease progression [25]. Nevertheless, the significant gains in social functioning suggest that patients experienced better social interactions and participation post-surgery.

Patients with benign lung nodules also showed significant QoL improvements, particularly in mental health domains. The relief from anxiety over potential malignancy and the confirmation of a benign diagnosis likely contributed to reduced psychological distress. This underscores the importance of addressing the psychological impact of uncertainty in patients with pulmonary nodules. The correlations between QoL improvements and clinical variables highlight important considerations. Younger patients and those with better baseline lung function experienced greater QoL gains, suggesting that patient selection is crucial for optimal outcomes. These findings emphasize the need for thorough preoperative evaluations to identify patients most likely to benefit from surgery.

Moreover, the study by Vallilo et al. [25] demonstrated that lung resection significantly improved the quality of life for patients with noncystic fibrosis bronchiectasis, with 53 patients achieving near-normal QoL scores on the Short Form-36 and WHO Quality of Life questionnaires by the 9-month follow-up. Initial assessments showed low QoL scores, mild lung obstruction, and reduced exercise capacity, but post-surgery, 52% of patients reported improved exercise performance despite a 24.5% rate of adverse events and a mild reduction in lung volume. Similarly, Magge et al. [26] reported that specialized care led to statistically significant improvements in Physical Functioning, Role Functioning, and Health Perceptions scores among bronchiectasis patients over a two-year period, with these improvements first noted at the one-year follow-up and sustained through the second year. These studies collectively highlight the effectiveness of targeted interventions in enhancing and sustaining life quality in bronchiectasis patients, whether through surgical or continuous specialized care approaches.

In a similar manner, the study by Pompili et al. [27] explored the quality of life outcomes over one year for patients undergoing video-assisted thoracoscopic surgery (VATS) and stereotactic ablative body radiotherapy (SABR) for non-small cell lung cancer (NSCLC). This longitudinal study included 225 patients, highlighting that those treated with SABR had significantly lower baseline QoL scores than the VATS group, with Global Health scores at 53.8 versus 71.2 and Physical Functioning at 57 versus 82.2, respectively. Over the year, SABR patients maintained stable QoL scores, whereas VATS patients experienced a decline in various QoL domains 6 weeks post-surgery, with some improvement by 12 months, yet not returning to preoperative levels. Conversely, Cansever et al. [28] conducted a retrospective analysis to compare the short-term QoL between patients undergoing VATS and those undergoing thoracotomy. With 96 participants analyzed, findings revealed that one month postoperatively, patients who underwent VATS reported better QoL outcomes compared to those who had thoracotomy, despite similar complication rates between the two groups (20.9% for VATS and 22.6% for thoracotomy). Both studies highlight the variable impact of different surgical interventions on patient QoL, with Pompili et al. showing a temporal deterioration in VATS patients’ QoL that partially recovers, and Cansever et al. demonstrating a more immediate QoL benefit for VATS over thoracotomy.

In the study by Brown et al. [29], the health-related quality of life (HRQOL) after lobectomy for lung cancer was explored through qualitative interviews with 25 patients, identifying key HRQOL domains including physical function, pain, fatigue, dyspnea, and emotional and social support. Nearly all participants reported concerns with general physical function, and significant numbers highlighted pain (96%), fatigue (96%), and dyspnea (76%). In addition, themes such as independence and preparation for surgery were notable. In a similar manner, the systematic review by Singer et al. [30] assessed the impact of different surgical approaches on QoL following lung cancer resection, comparing minimally invasive techniques like VATS and robotic-assisted thoracic surgery with open thoracotomy. This review included 15 studies and found that minimally invasive approaches generally resulted in better QoL outcomes, especially in physical functioning (reported by 9 out of 14 studies) and pain management (7 out of 12 studies). 

The findings from the study underscore the significant clinical utility of assessing and comparing quality of life and psychological outcomes across different patient groups undergoing thoracic surgery. Notably, the TB group showed the most substantial improvements in both physical and mental health post-surgery, as indicated by SF-36, WHOQOL-BREF, and HADS scores, with statistically significant better outcomes compared to the bronchiectasis and benign nodules groups. These results highlight the potential for tailored preoperative evaluations and targeted postoperative care plans based on patient-specific factors such as age and baseline lung function (FEV1), which are correlated with better recovery outcomes. This approach can improve patient counseling on expected post-surgical recovery and aid clinicians in optimizing treatment strategies to enhance overall patient well-being after surgery.

Nevertheless, this study brings novelty to the literature by systematically comparing the quality of life outcomes among patients undergoing pulmonary lobe resection for three different benign diseases: tuberculosis, bronchiectasis, and benign lung nodules. Such comparative analysis is rare, particularly the inclusion of a tuberculosis cohort, which introduces unique insights into the specific benefits of surgical intervention for this group. This approach not only underscores the variability in recovery and quality of life improvements across different conditions but also highlights the profound impact that surgery can have on tuberculosis patients, providing valuable data that could influence both future research and clinical practice.

### 4.2. Study Limitations and Future Perspectives

This study has several limitations. First, the sample size, while adequate for initial analyses, may limit the generalizability of the findings. This sample size also restricts the ability to effectively adjust for multiple covariates and may compromise the statistical power and accuracy in controlling for potential confounders, increasing the risk of overfitting and misleading conclusions. Larger, multicenter studies are needed to confirm these results and enhance external validity. Second, the follow-up period of 6 months may not capture long-term QoL outcomes or late complications. Future studies with extended follow-up are necessary to assess the durability of observed improvements. Third, the reliance on self-reported questionnaires for QoL and psychological assessments may introduce response bias. Incorporating objective measures, such as physical performance tests and clinical evaluations, could provide a more comprehensive assessment. Additionally, the study did not account for potential socioeconomic factors or variations in access to postoperative rehabilitation services, which may influence QoL outcomes. Finally, the observational design and lack of randomization may introduce selection bias and confounding factors despite efforts to control for baseline characteristics. Randomized controlled trials are needed to establish causal relationships between surgical intervention and QoL improvements.

Future studies could consider increasing the sample size to allow for more robust multivariable analysis. This would enable researchers to better isolate the effects of the surgical intervention from other influencing factors such as demographic and clinical characteristics.

## 5. Conclusions

Pulmonary lobe resection for benign diseases significantly enhances quality of life and psychological well-being over a 6-month period, with the most substantial improvements observed in patients with tuberculosis. These findings suggest that surgical intervention not only alleviates physical symptoms but also has a positive impact on mental health. Clinicians should consider QoL outcomes when recommending surgery for benign lung diseases and provide comprehensive preoperative counseling. Patient selection should involve assessing factors such as age and baseline lung function to optimize postoperative outcomes. Future research should focus on long-term QoL assessment and explore strategies to further enhance patient care, including postoperative rehabilitation and psychological support.

## Figures and Tables

**Table 1:** Demographic and clinical characteristics of patients.

Variable	TB Group (n = 22)	Bronchiectasis Group (n = 31)	Benign Nodules Group (n = 31)	*p*-Value
Age (years)	54.2 ± 9.1	56.8 ± 8.5	55.7 ± 7.9	0.512
Gender (Male/Female)	14/8	18/13	19/12	0.891
Smoking Status (%)				0.603
Current Smokers	10 (45%)	13 (42%)	12 (39%)	0.927
Ex-Smokers	6 (27%)	9 (29%)	10 (32%)	
Non-Smokers	6 (27%)	9 (29%)	9 (29%)	
Comorbidities (%)			0.823	0.462
Hypertension	5 (23%)	7 (23%)	6 (19%)	
Diabetes Mellitus	3 (14%)	4 (13%)	5 (16%)	
FEV1 (% predicted)	78.5 ± 12.3	76.9 ± 11.8	77.8 ± 10.5	0.814

TB—Tuberculosis; FEV—Forced Expiratory Volume.

**Table 2:** Baseline QoL and psychological scores preoperatively.

Variable	TB Group (n = 22)	Bronchiectasis Group (n = 31)	Benign Nodules Group (n = 31)	*p*-Value
SF-36 Scores				0.512
-Physical Component Summary (PCS)	41.2 ± 6.5	43.1 ± 7.0	42.5 ± 6.8	0.512
Mental Component Summary (MCS)	38.7 ± 7.2	40.0 ± 6.9	39.5 ± 7.1	0.678
WHOQOL-BREF Scores				0.927
Physical Health	52.4 ± 9.1	54.8 ± 8.7	53.9 ± 8.5	0.612
Psychological	50.2 ± 8.9	51.5 ± 9.2	51.0 ± 9.0	0.798
Social Relationships	49.5 ± 7.8	50.8 ± 8.1	50.2 ± 8.0	0.829
Environment	55.1 ± 8.3	56.3 ± 8.5	55.9 ± 8.4	0.876
HADS Scores				
Anxiety	10.5 ± 3.2	10.0 ± 3.5	10.2 ± 3.3	0.821
Depression	9.8 ± 2.9	9.5 ± 3.1	9.6 ± 3.0	0.915
PSS-10 Score	21.5 ± 4.5	20.8 ± 4.8	21.0 ± 4.6	0.772

TB—Tuberculosis; SF—Short Form; WHO—World Health Organization; PSS—Perceived Stress Scale; HADS—Hospital Anxiety and Depression Scale; QOL—Quality of Life.

**Table 3:** QoL and psychological scores at 6 months after surgery.

Variable	TB Group (n = 22)	Bronchiectasis Group (n = 31)	Benign Nodules Group (n = 31)	*p*-Value
SF-36 Scores				0.512
PCS	52.8 ± 5.0	49.5 ± 6.2	49.0 ± 6.5	0.013
MCS	50.2 ± 6.1	47.8 ± 6.8	48.0 ± 6.7	0.045
WHOQOL-BREF Scores				0.927
Physical Health	68.5 ± 7.8	62.3 ± 8.5	61.8 ± 8.7	0.004
Psychological	66.2 ± 8.0	60.5 ± 8.3	60.0 ± 8.5	0.011
Social Relationships	65.0 ± 7.5	63.8 ± 7.9	62.5 ± 8.0	0.428
Environment	70.2 ± 8.1	68.5 ± 8.4	68.0 ± 8.6	0.571
HADS Scores				
Anxiety	6.2 ± 2.5	7.8 ± 2.8	7.5 ± 2.7	0.022
Depression	5.5 ± 2.3	6.9 ± 2.6	6.7 ± 2.5	0.037
PSS-10 Score	14.8 ± 3.9	17.2 ± 4.1	17.5 ± 4.3	0.019

TB—Tuberculosis; SF—Short Form; WHO—World Health Organization; PSS—Perceived Stress Scale; HADS—Hospital Anxiety and Depression Scale; QOL—Quality of Life.

**Table 4:** Within-group comparisons of preoperative and postoperative scores.

Variable	Preoperative Mean ± SD	Postoperative Mean ± SD	Mean Difference ± SD	*p*-Value
TB Group (n = 22)				
SF-36 PCS	41.2 ± 6.5	52.8 ± 5.0	+11.6 ± 4.2	<0.001
SF-36 MCS	38.7 ± 7.2	50.2 ± 6.1	+11.5 ± 4.5	<0.001
HADS Anxiety	10.5 ± 3.2	6.2 ± 2.5	−4.3 ± 2.1	<0.001
HADS Depression	9.8 ± 2.9	5.5 ± 2.3	−4.3 ± 2.0	<0.001
PSS-10	21.5 ± 4.5	14.8 ± 3.9	−6.7 ± 2.5	<0.001
Bronchiectasis Group (n = 31)				
SF-36 PCS	43.1 ± 7.0	49.5 ± 6.2	+6.4 ± 3.8	<0.001
SF-36 MCS	40.0 ± 6.9	47.8 ± 6.8	+7.8 ± 4.1	<0.001
HADS Anxiety	10.0 ± 3.5	7.8 ± 2.8	−2.2 ± 1.7	<0.001
HADS Depression	9.5 ± 3.1	6.9 ± 2.6	−2.6 ± 1.8	<0.001
PSS-10	20.8 ± 4.8	17.2 ± 4.1	−3.6 ± 2.2	<0.001
Benign Nodules Group (n = 31)				
SF-36 PCS	42.5 ± 6.8	49.0 ± 6.5	+6.5 ± 3.9	<0.001
SF-36 MCS	39.5 ± 7.1	48.0 ± 6.7	+8.5 ± 4.3	<0.001
HADS Anxiety	10.2 ± 3.3	7.5 ± 2.7	−2.7 ± 1.8	<0.001
HADS Depression	9.6 ± 3.0	6.7 ± 2.5	−2.9 ± 1.9	<0.001
PSS-10	21.0 ± 4.6	17.5 ± 4.3	−3.5 ± 2.1	< 0.001

TB—Tuberculosis; SF—Short Form; WHO—World Health Organization; PSS—Perceived Stress Scale; HADS—Hospital Anxiety and Depression Scale; QOL—Quality of Life.

**Table 5:** Correlation of QoL improvement with clinical variables.

Variable	Correlation Coefficient (r)	*p*-Value
Age vs. SF-36 PCS Improvement	−0.35	0.001
FEV1 (% predicted) vs. SF-36 PCS Improvement	0.42	<0.001
Smoking Status vs. SF-36 PCS Improvement	−0.1	0.341
Comorbidities vs. SF-36 PCS Improvement	−0.15	0.181

**Table 6:** Postoperative complications and outcomes.

Variable	TB Group (n = 22)	Bronchiectasis Group (n = 31)	Benign Nodules Group (n = 31)	*p*-Value
Postoperative Complications (%)	2 (9%)	4 (13%)	3 (10%)	0.815
Prolonged Air Leak	1 (4.5%)	2 (6.5%)	1 (3.2%)	
Wound Infection	1 (4.5%)	1 (3.2%)	1 (3.2%)	
Pneumonia	0 (0%)	1 (3.2%)	1 (3.2%)	
Length of Hospital Stay (days)	7.5 ± 1.8	6.8 ± 1.5	6.5 ± 1.4	0.089
30-day Mortality (%)	0 (0%)	0 (0%)	0 (0%)	-

TB—Tuberculosis; SF—Short Form; WHO—World Health Organization; PSS—Perceived Stress Scale; HADS—Hospital Anxiety and Depression Scale; QOL—Quality of Life.

## Data Availability

Data are available on request.

## References

[1] Pompili C. (2015). Quality of life after lung resection for lung cancer. J. Thorac. Dis..

[2] Villar-Hernández R., Ghodousi A., Konstantynovska O., Duarte R., Lange C., Raviglione M. (2023). Tuberculosis: Current challenges and beyond. Breathe.

[3] Calligaro G.L., Moodley L., Symons G., Dheda K. (2014). The medical and surgical treatment of drug-resistant tuberculosis. J. Thorac. Dis..

[4] Tseng Y.L., Chang J.M., Liu Y.S., Cheng L., Chen Y.Y., Wu M.H., Lu C.L., Yen Y.T. (2016). The Role of Video-Assisted Thoracoscopic Therapeutic Resection for Medically Failed Pulmonary Tuberculosis. Medicine.

[5] King P.T. (2009). The pathophysiology of bronchiectasis. Int. J. Chronic Obstruct Pulm. Dis..

[6] Feng J., Sun L., Sun X., Xu L., Liu L., Liu G., Wang J., Gao P., Zhan S., Chen Y. (2022). Increasing prevalence and burden of bronchiectasis in urban Chinese adults, 2013-2017: A nationwide population-based cohort study. Respir. Res..

[7] Gülhan S.Ş.E., Acar L.N., Sayılır Güven E., Bıçakçıoğlu P., Aydın E., Karasu S., Taştepe A.İ., İncekara F., Kaya S., Fındık G. (2020). Surgical treatment of bronchiectasis: Our 23 years of experience. Turk. Gogus Kalp Damar Cerrahisi Derg..

[8] Schmid-Bindert G., Vogel-Claussen J., Gütz S., Fink J., Hoffmann H., Eichhorn M.E., Herth F.J.F. (2022). Incidental Pulmonary Nodules—What Do We Know in 2022. Respiration.

[9] Loverdos K., Fotiadis A., Kontogianni C., Iliopoulou M., Gaga M. (2019). Lung nodules: A comprehensive review on current approach and management. Ann. Thorac. Med..

[10] Wolyniec K., Sharp J., Fisher K., Tothill R.W., Bowtell D., Mileshkin L., Schofield P. (2022). Psychological distress, understanding of cancer and illness uncertainty in patients with Cancer of Unknown Primary. Psychooncology.

[11] Artinyan A., Orcutt S.T., Anaya D.A., Richardson P., Chen G.J., Berger D.H. (2015). Infectious postoperative complications decrease long-term survival in patients undergoing curative surgery for colorectal cancer: A study of 12,075 patients. Ann. Surg..

[12] Lv X., Cao J., Dai X., Rusidanmu A. (2018). Survival rates after lobectomy versus sublobar resection for early-stage right middle lobe non-small cell lung cancer. Thorac. Cancer.

[13] Galata C., Karampinis I., Roessner E.D., Stamenovic D. (2021). Risk factors for surgical complications after anatomic lung resections in the era of VATS and ERAS. Thorac. Cancer.

[14] Nicola A., Oancea C., Barata P.I., Adelina M., Mateescu T., Manolescu D., Bratosin F., Fericean R.M., Pingilati R.A., Paleru C. (2023). Health-Related Quality of Life and Stress-Related Disorders in Patients with Bronchiectasis after Pulmonary Resection. J. Pers. Med..

[15] Nouri F., Feizi A., Roohafza H., Sadeghi M., Sarrafzadegan N. (2021). How different domains of quality of life are associated with latent dimensions of mental health measured by GHQ-12. Health Qual. Life Outcomes.

[16] Trippoli S., Vaiani M., Lucioni C., Messori A. (2001). Quality of life and utility in patients with non-small cell lung cancer. Quality-of-life Study Group of the Master 2 Project in Pharmacoeconomics. Pharmacoeconomics.

[17] Liang W.M., Chen J.J., Chang C.H., Chen H.W., Chen S.L., Hang L.W., Wang J.D. (2008). An empirical comparison of the WHOQOL-BREF and the SGRQ among patients with COPD. Qual. Life Res..

[18] Pompili C., Trevis J., Patella M., Brunelli A., Libretti L., Novoa N., Scarci M., Tenconi S., Dunning J., Cafarotti S. (2021). European Society of Thoracic Surgeons electronic quality of life application after lung resection: Field testing in a clinical setting. Interact. Cardiovasc. Thorac. Surg..

[19] Shrestha B., Dunn L. (2020). The Declaration of Helsinki on Medical Research involving Human Subjects: A Review of Seventh Revision. J. Nepal Health Res. Counc..

[20] Michopoulos I., Douzenis A., Kalkavoura C., Christodoulou C., Michalopoulou P., Kalemi G., Fineti K., Patapis P., Protopapas K., Lykouras L. (2008). Hospital Anxiety and Depression Scale (HADS): Validation in a Greek general hospital sample. Ann. Gen. Psychiatry.

[21] Xiao T., Zhu F., Wang D., Liu X., Xi S.J., Yu Y. (2023). Psychometric validation of the Perceived Stress Scale (PSS-10) among family caregivers of people with schizophrenia in China. BMJ Open.

[22] Vashakidze S.A., Kempker J.A., Jakobia N.A., Gogishvili S.G., Nikolaishvili K.A., Goginashvili L.M., Magee M.J., Kempker R.R. (2019). Pulmonary function and respiratory health after successful treatment of drug-resistant tuberculosis. Int. J. Infect. Dis..

[23] Salami M.A., Sanusi A.A., Adegboye V.O. (2018). Current Indications and Outcome of Pulmonary Resections for Tuberculosis Complications in Ibadan, Nigeria. Med. Princ. Pract..

[24] Hasan E.M., Calma C.L., Tudor A., Oancea C., Tudorache V., Petrache I.A., Tudorache E., Papava I. (2021). Coping, Anxiety, and Pain Intensity in Patients Requiring Thoracic Surgery. J. Pers. Med..

[25] Vallilo C.C., Terra R.M., de Albuquerque A.L., Suesada M.M., Mariani A.W., Salge J.M., da Costa P.B., Pêgo-Fernandes P.M. (2014). Lung resection improves the quality of life of patients with symptomatic bronchiectasis. Ann. Thorac. Surg..

[26] Magge A., Ashraf S., Quittner A.L., Metersky M.L. (2019). Quality of life in patients with bronchiectasis: A 2-year longitudinal study. Ann. Transl. Med..

[27] Pompili C., Rogers Z., Absolom K., Holch P., Clayton B., Callister M., Robson J., Brunelli A., Franks K., Velikova G. (2021). Quality of life after VATS lung resection and SABR for early-stage non-small cell lung cancer: A longitudinal study. Lung Cancer.

[28] Cansever L., Sezen C.B., Yaran O.V., Bedirhan M.A. (2020). Comparison of short-term quality of life in patients undergoing video-assisted thoracoscopic surgery versus thoracotomy. Turk. Gogus Kalp Damar Cerrahisi Derg..

[29] Brown L.M., Gosdin M.M., Cooke D.T., Apesoa-Varano E.C., Kratz A.L. (2020). Health-Related Quality of Life After Lobectomy for Lung Cancer: Conceptual Framework and Measurement. Ann. Thorac. Surg..

[30] Singer E.S., Kneuertz P.J., Nishimura J., D’Souza D.M., Diefenderfer E., Moffatt-Bruce S.D., Merritt R.E. (2020). Effect of operative approach on quality of life following anatomic lung cancer resection. J. Thorac. Dis..

